# Intravascular Lithotripsy for Patients with Chronic Limb-Threatening Ischemia: Study Protocol for CALCIO, a Prospective Multicenter Observational Investigation

**DOI:** 10.1007/s00270-025-04335-w

**Published:** 2026-02-17

**Authors:** Raman Uberoi, Peter Reimer, Joo-Young Chun, Gilles Goyault, Elika Kashef, Romaric Loffroy, Thomas Rand, Maria Antonella Ruffino, Conrad von Stempel, Tilmann Lange, Bleranda Zeka, Claire Poulet, Nathalie Kaufmann, Christoph Binkert

**Affiliations:** 1 Department of Radiology, John Radcliffe Hospital, Oxford University Hospitals NHS Trust, Headington, Oxford, OX3 9DU UK; 2 Institute of Diagnostic and Interventional Radiology, Klinikum Karlsruhe, Moltkestraße 90, 76133 Karlsruhe, Germany; 3 Department of Radiology, St George’s University Hospitals NHS Foundation Trust, Blackshaw Rd, London, SW17 0QT UK; 4 Cardiovascular Institute of Strasbourg, Clinique Rhéna de Strasbourg, 10 Rue François Epailly, 67000 Strasbourg, France; 5 Department of Imaging, Imperial College Healthcare NHS Trust, The Bays, S Wharf Rd, London, W2 1NY UK; 6 Department of Vascular and Interventional Radiology, François-Mitterrand University Hospital, 14 Rue Gaffarel, 21079 Dijon Cedex, France; 7 Institute of Radiology, Klinik Floridsdorf, Brünner Straße 68, 1210 Vienna, Austria; 8 Interventional Radiology, Imaging Institute of Southern Switzerland, EOC Lugano, Via Tesserete 46, 6900 Lugano, Switzerland; 9 Division of Surgery and Interventional Science, UCL, London, UK; 10 UCLH NHS Foundation Trust, 235 Euston Road, London, NW1 2BU UK; 11 Royal Free London NHS Foundation Trust, Pond Street, London, NW3 2QG UK; 12 Next Research, Cardiovascular and Interventional Radiological Society of Europe, Neutorgasse 9, 1010 Vienna, Austria; 13 Interventional Radiology at Medical Radiology Institute Zurich, Hofwiesenstrasse 349, 8050 Zurich, Switzerland

**Keywords:** Chronic limb-threatening ischemia, Intravascular lithotripsy, Peripheral arterial disease, Vascular calcification, Endovascular treatment

## Abstract

**Purpose:**

The prevalence of severe vascular calcification in patients with Chronic Limb-Threatening Ischemia (CLTI) hinders the outcome of endovascular interventions and increases the risk of amputation and death. Intravascular lithotripsy (IVL) is a safe and effective treatment that promotes revascularization; however, large-scale data on its long-term effectiveness are lacking. The Chronic Limb-Threatening Ischemia treated with Intravascular Lithotripsy Observational Study (CALCIO) aims to address this shortcoming by assessing the real-world, long-term outcomes of IVL in a large cohort of patients with CLTI.

**Methods:**

CALCIO is a worldwide, multicenter, prospective cohort study designed to follow for 2 years 400 participants treated with the Shockwave Medical IVL System. Eligible patients, adults (≥ 18 years) with CLTI (Rutherford category ≥ 4) and calcified femoro-popliteal and/or crural lesions visible on fluoroscopy, are recruited upon providing informed consent. The primary outcome measure is a composite of wound healing and freedom from amputation at 12 months. Secondary outcome measures include technical success, amputation-free survival, changes in Rutherford category, limb perfusion and wound, ischemia, and foot infection (WIfI) score, patency, need for revascularization, safety, and patient-reported health-related quality of life (HRQoL). Results will be reported using mainly descriptive statistics. Patient enrollment opened in July 2024 and is planned to continue until July 2026, with the follow-up period expected to end in July 2028.

**Conclusion:**

CALCIO intends to provide valuable insights into the real-world long-term clinical outcomes of IVL to support decision making regarding the optimal endovascular treatment for CLTI patients. CALCIO is registered on ClinicalTrials.gov (NCT06149650).

**Graphical Abstract:**

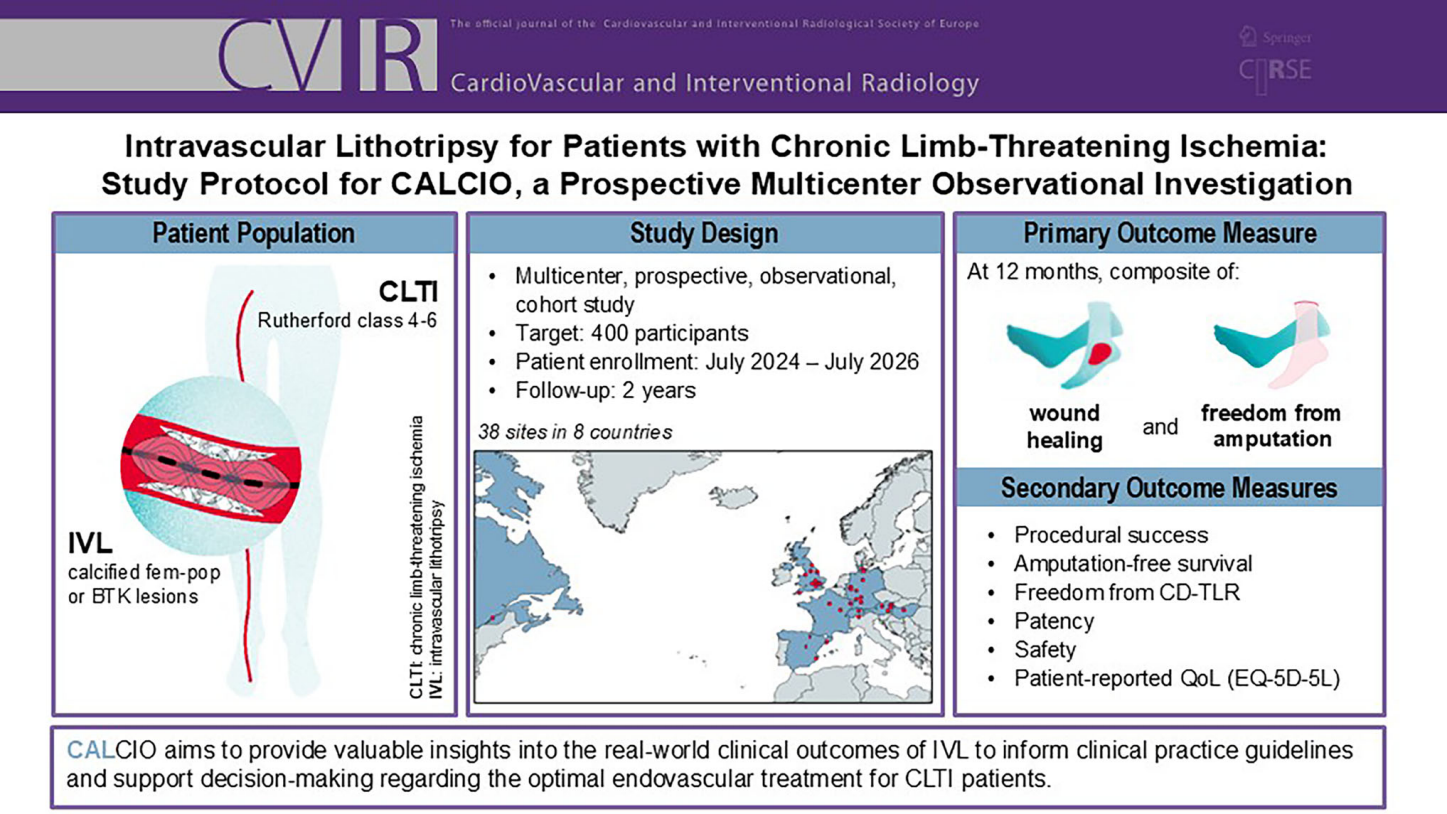

## Introduction

Peripheral arterial disease (PAD) affects more than 100 million people worldwide and is associated with significant morbidity, mortality, and health-related quality of life (HRQoL) impairment [1, 2]. Endovascular interventions have become the primary revascularization strategy for PAD; however, the presence of vascular calcification reduces the effectiveness of many of these procedures by restricting vessel wall expansion, hindering drug absorption, and increasing the risk for procedural complications such as vascular dissection, stent fracture, and stent thrombosis [3–7]. In fact, severe calcification is a significant predictor of poor technical success and unfavorable clinical outcomes of endovascular treatments [8–11]. This is especially problematic for patients with Chronic Limb-Threatening Ischemia (CLTI), who show a high prevalence of severe, often circumferential femoro-popliteal and crural calcifications [11–13].

Intravascular lithotripsy (IVL) has been developed specifically to address these challenges using specialized balloon catheters that deliver sonic pressure waves to disrupt calcium deposits and thereby improve vessel compliance and revascularization outcomes. The technique’s safety and efficacy in achieving significant luminal gain have been demonstrated by two recent systematic reviews and meta-analyses, which reported an overall 55–60% reduction in diameter stenosis and low rates of periprocedural complications (~ 1.2%) [14, 15]. Despite these promising preliminary results, data on the long-term effectiveness of IVL remain scarce, particularly in CLTI patients.

The Chronic Limb-Threatening Ischemia treated with Intravascular Lithotripsy Observational Study (CALCIO) has been designed to address these important gaps in our understanding of the real-world performance of IVL for PAD by i) focusing specifically on patients with CLTI; ii) collecting 2-year follow-up data; iii) looking primarily at objective clinical outcomes that directly reflect patient quality of life (i.e., wound healing and freedom from amputation); and iv) supplementing these observations with patient-reported outcomes.

## Study Design and Site Selection

CALCIO is a prospective, multicenter, single-cohort, observational study. It is sponsored by the Cardiovascular and Interventional Radiological Society of Europe (CIRSE) and governed by a scientific Steering Committee composed of interventional radiologists with extensive expertise in the field of PAD. The study is conducted in compliance with the Declaration of Helsinki, good clinical practice, and all relevant European and national regulations and has received all necessary approvals from national or local ethics committees and regulatory authorities, if appropriate.

Sites in Europe and North America with expertise in the management of CLTI patients (≥ 50 CLTI patients treated per year) and experience with the Shockwave Medical IVL System (Shockwave Medical, Inc., California, USA; ≥ 10 cases to date) were invited to participate in the study. Approximately 40 sites across 9 countries (Austria, Canada, France, Germany, Hungary, Italy, Spain, Switzerland, the UK) are expected to contribute to the study.

## Study Population

A total of 400 patients with CLTI receiving IVL per standard of care will be recruited upon providing informed consent. Eligibility criteria are listed in Table 1. Principal investigators (PIs) are instructed to present the possibility to participate in CALCIO to all eligible patients and document refusal and reasons for non-eligibility when appropriate.

**Table 1:** Patient inclusion and exclusion criteria

Inclusion criteria
1. Patient with Chronic Limb-Threatening Ischemia (Rutherford classification category ≥ 4)
2. Femoro-popliteal and/or crural calcified lesions visible on fluoroscopy
3. Treatment using the Shockwave Medical Intravascular Lithotripsy System

Exclusion criteria
1. < 18 years old
2. Incapacity or refusal to give informed consent
3. Ongoing pregnancy
4. Endovascular procedure(s) on the treatment site within 4 weeks before the planned intravascular lithotripsy treatment

Areas treated with IVL are referred to as “target segments,” and a segment is defined as a contiguous diseased area with less than 5 cm separation. Since there are no treatment restrictions, IVL may be performed on one or multiple segments, alone or with adjunctive endovascular interventions, on one or both limbs, on the same day or over multiple sessions. Patients may also receive endovascular or surgical procedures on non-target segments.

## Data Collection and Quality Control

**Fig. 1:**
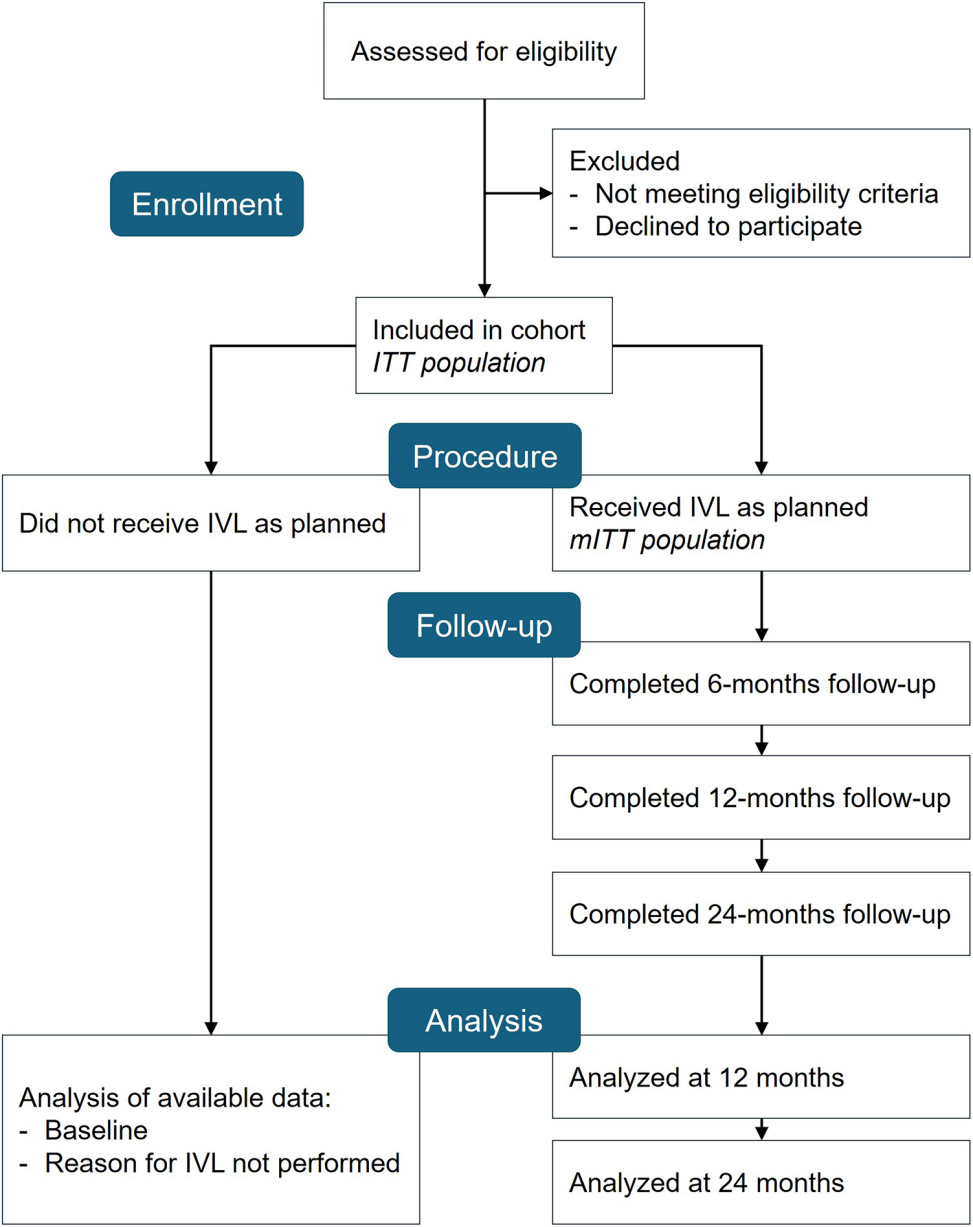
Flowchart illustrating the participant pathway and corresponding data analysis. ITT, intent-to-treat; mITT, modified intent-to-treat

Baseline data, obtained within 6 weeks before the first planned IVL treatment, include demographics (age and sex) and relevant medical history (smoking habit, diabetes, hypertension, hyperlipidemia, symptomatic ischemic heart disease, ongoing anti-platelet/anti-coagulation treatment). Data specific to the target limb, including the presence and extent of ischemic ulcers and foot infection, measurement of limb perfusion (ankle–brachial index, ankle systolic pressure, toe pressure, or transcutaneous oximetry), and history of prior surgical or endovascular treatments for PAD, as well as data specific to the target segments, such as location, degree of stenosis, and length of occlusion (if relevant), are also captured. Upon completion of the first IVL treatment, procedural details, including information on any potential adjunctive intervention, are collected for each target segment together with digital subtraction angiography (DSA) images and non-contrast images taken from the unsubtracted mask of DSA images. In case patients do not receive IVL as planned, reasons for not proceeding with the planned treatment are recorded, and data collection is suspended (Fig. 1). For all treated patients, follow-up data are collected at 6, 12, and 24 months. Safety data are collected from the first IVL procedure throughout the study, and HRQoL is planned to be captured at each visit.

All data points and images are collected via an electronic data capture (EDC) system provided by REDCap Cloud (nPhase, Inc, version 1.7.3) using electronic case report forms (eCRFs) which contain constraints on data types and ranges, as well as alerts for inconsistent or missing data entry to ensure data validity, completeness, consistency, and uniformity. Additionally, further consistency checks are performed using data exports. All open queries are tracked and summaries are sent to the sites regularly. A regular remote monitoring scheme is employed for each participating site to verify data quality and identify potential issues regarding patient enrollment and data collection. While no on-site monitoring or source data verification is planned due to resource limitations, a Blinded Central Image Review (BCIR) will be used to assess selected outcome measures.

## Outcome measures

A list of all outcome measures is summarized in Table 2.

**Table 2:** Primary and secondary outcome measures in CALCIO

	Outcome measure	When	Reported by
Effectiveness—Primary	Composite of wound healing and freedom from amputation	12 months	Investigator
Effectiveness—Secondary	Technical success of IVL (diameter stenosis ≤ 30% after IVL)	Treatment day	BCIR
	Overall procedural success (diameter stenosis ≤ 30% after complete treatment in case of adjunctive interventions)	Treatment day	BCIR
	Wound healing	12 and 24 months	Investigator
	Freedom from amputation	12 and 24 months	Investigator
	Amputation-free survival	Time-to-event	Investigator
	Change in Rutherford category	12 and 24 months	Investigator
	Change in limb perfusion (based on ankle–brachial index, ankle systolic pressure, toe pressure, or transcutaneous oximetry)	12 and 24 months	Investigator
	Change in WIfI score	12 and 24 months	Investigator
	CD-TLR	12 and 24 months	Investigator
	CD-TLR-free survival	Time-to-event	Investigator
	Patency (primary, assisted primary, secondary)	12 and 24 months	Investigator
Safety	Incidence and severity of procedural complications and major AEs	Within 30 days after IVL	Investigator
HRQoL	EuroQol EQ-5D-5L questionnaire	6, 12, and 24 months	Patient
Exploratory	Change in vascular calcification	Treatment day	BCIR

IVL, intravascular lithotripsy; WIfI score, wound; infection, and foot infection score; CD-TLR, clinically driven target lesion revascularization; AEs, adverse events; HRQoL, health-related quality of life; BCIR, blinded central image review

### Primary Outcome Measure

The primary outcome measure of CALCIO is a composite of wound healing and freedom from amputation at 12 months after the first IVL procedure. Wound healing is defined as the healing status of the dominant ischemic wound of the target limb as assessed by the investigators using the categories defined in Table 3.

**Table 3:** Definitions of wound healing status

Wound healing	
Complete	Complete epithelialization of the wound persistent for at least 14 days
Partial	Decrease of wound size > 50% compared to baseline (in largest diameter)
Unchanged	Increase or decrease of wound size ≤ 50% compared to baseline (in largest diameter)
Worsening	Increase of wound size > 50% compared to baseline (in largest diameter)

Patients who presented no wound at baseline (Rutherford category 4) are followed for the development of de novo ischemic ulcers. If such an ulcer appears after treatment, the limb will be reported separately as “worsening.”

Freedom from amputation will be assessed by considering both minor (digital, metatarsal) and major (below or above the knee) amputations; however, amputations that had been planned before the first IVL treatment will be reported separately. For patients who receive an amputation, healing of the stump will be reported.

### Secondary Outcome Measures

#### Immediate Effectiveness

Immediate effectiveness, per treated segment, is defined as residual diameter stenosis ≤ 30% and will be assessed by BCIR using DSA images at the following time points: 1) after IVL and before any potential adjunctive intervention, “**technical success of IVL,**” and 2) by the end of the complete procedure after any potential, planned or unplanned, adjunctive intervention, “**overall procedural success.**”

#### Long-Term Effectiveness

To further assess the long-term effectiveness of IVL, **wound healing** and **freedom from amputation**, as defined above, will be reassessed at 24 months, and **amputation-free survival** will be reported from the time of the first IVL treatment, excluding patients with any amputation that had been planned beforehand.

Other secondary effectiveness outcome measures that will be assessed at 12 and 24 months include: i) changes of Rutherford category, limb perfusion, and WIfI score [16–18]; ii) **freedom from clinically driven target lesion revascularization (CD-TLR)**, defined as the absence of any endovascular re-intervention to the target segment (± 10 mm) or surgical bypass performed because of restenosis or occlusion and **CD-TLR-free survival**; and iii) patency of the target segments with a distinction between **primary patency** (absence of total occlusion without any re-intervention to the target segment); **assisted primary patency** (absence of total occlusion following re-intervention on the target segment due to restenosis); and **secondary patency** (absence of total occlusion following re-intervention on the target segment due to occlusion).

#### Safety

Safety of IVL will be assessed by monitoring the incidence and severity of major adverse events (AEs) within 30 days after the procedure. These include, but are not limited to, procedural complications at the target and access sites (flow-limiting dissection, emboli, occlusion, infection, hematoma, false aneurysm, and perforation); deterioration of renal function; allergic reaction; sepsis; acute onset of limb ischemia; stroke and myocardial infarction. Severity will be graded according to the CIRSE classification system [19]. The total number of AEs and the percentage of patients with at least one AE will be summarized for AEs of i) any grade, ii) grade 3–4, and iii) grade 5. A further distinction will be made based on the likelihood of a causal relationship between the AE and the IVL procedure, as assessed by the PIs.

#### Patient-Reported Health-Related Quality of Life

Patient-reported HRQoL will be evaluated using the EuroQol questionnaire EQ-5D-5L. This questionnaire includes 5 dimensions (mobility, self-care, usual activities, pain or discomfort, and anxiety or depression), each having 5 response levels (no problems, slight problems, moderate problems, severe problems, unable to/extreme problems) as well as a vertical visual analog scale (EQ VAS) from 1 to 100 for the patients to rate their general health. The number and percentages of responses per dimension will be analyzed and reported together with the mean, standard deviation, median, maximum, and minimum values of the EQ VAS.

#### Change in Vascular Calcification

The visualization and quantification of potential changes in local vascular calcification immediately following IVL will be investigated as an exploratory outcome. For this purpose, non-contrast images of the target segments taken from unsubtracted mask of DSA images pre- and post-IVL will be collected and analyzed by BCIR.

## Statistical Analysis

CALCIO is a single-arm study that will use mainly descriptive statistics to report the outcome measures. The target of 400 participants was not determined through a formal sample size calculation but was assessed by the Steering Committee as a sufficiently large sample to provide meaningful information on the treatment’s effectiveness while remaining achievable within the planned enrollment period. All participants who were planned to receive IVL will be included in the intent-to-treat (ITT) population, regardless of whether they received the planned procedure. In contrast, the modified intent-to-treat (mITT) population will only include participants who received IVL treatment.

The results of the primary outcome measure will be presented using summary statistics and a 95% confidence interval (CI). For time-to-event outcome measures (amputation-free survival) and (CD-TLR-free survival), median time-to-event and 95% CI will be calculated using the Kaplan–Meier estimator, while the impact of different covariates will be assessed using a stratified log-rank test and an univariable Cox proportional hazard regression. Following that, covariates with *p* values < 0.1 and ≤ 20% missing data will be included in a multivariable Cox proportional hazards regression model, while variables with p values < 0.05 will be retained in the model after backward elimination or if they contribute to the predictability of the model. All variables that were not included in the model due to extensive missing data will be listed. All other statistical tests will be based on a significance level of 5%, and estimates will be presented using 95% bilateral CIs. No adjustment for multiple testing will be done. In the analyses of time-to-event outcomes, data will be censored at the last time the patients’ status was assessed.

In general, no imputation of missing data is planned for the analyses, and missing observations will be presented as a percentage of the total. However, for the primary outcome measure, depending on the number of deceased and lost-to-follow-up participants before 12 months, a sensitivity analysis may be performed.

Given the absence of planned confirmatory hypothesis testing, findings should be considered exploratory. The statistical analysis will be conducted using the latest available versions of R and RStudio.

## Discussion

IVL is a relatively recent technology with an expanding evidence base in claudicant patients demonstrating a favorable safety profile and significant luminal gain in calcified lesions; however, evidence in patients with CLTI remains limited. A recent single-center retrospective study on CLTI patients receiving IVL plus drug-coated balloon reported promising 12-month outcomes, with 92% freedom from CD-TLR, 98% freedom from major amputation, and 89% overall survival [20].

Despite the consensus that vascular calcification hinders clinical outcomes of endovascular interventions, European and American guidelines on the management of PAD offer limited, non-specific direction on revascularization strategies for severely calcified lesions and do not consistently address IVL or other plaque-modifying techniques [1, 2]. Reimbursement for IVL remains variable across healthcare systems, which likely constrains routine adoption.

In the UK, however, the National Institute for Health and Care Excellence (NICE) released its guidance on IVL for calcified arteries in PAD in 2024 (www.nice.org.uk/guidance/ipg780). Acknowledging “an unmet need for a safe and effective endovascular option for treating heavily calcified arterial lesions when surgery is unsuitable,” the NICE recommendations state that IVL can be used with special arrangements for clinical governance, consent, and audit or research. The existence of such guidelines is directly reflected in clinical practice, as a larger number of patients have access to the procedure in the UK compared to other countries. However, while the NICE guidance is based on the conclusion that IVL is safe and associated with a reduced need for stenting, it also highlights a lack of evidence regarding long-term patient outcomes. NICE specifically encourages further research to include details on patient selection (including lesion location and stenosis severity), adjunctive interventions, the need for repeat revascularization, patient-reported quality of life, amputation, and mortality.

Designed to specifically address these evidence gaps, CALCIO intends to complement the existing literature by collecting prospective data on the use of IVL in real-world CLTI practice. The findings are expected to contribute to future discussions on the role of IVL in clinical guidelines and inform reimbursement considerations for its use in CLTI patients. The study was received very positively by the community, with over 50 sites expressing interest in participating.

Patient enrollment for CALCIO opened in July 2024 and is planned to continue until July 2026. The follow-up period is expected to end in July 2028. At the time of manuscript submission, 38 sites from 8 countries, including 14 in the UK, were participating in the study and 357 patients had been enrolled, including 182 in the UK.

## Conclusions

CALCIO is the first large-scale study on the use of IVL for patients with CLTI. By collecting robust real-world data on clinical outcomes that are crucial to patients, in particular wound healing, freedom from amputation, and quality of life, CALCIO aims to provide pivotal information on the long-term impact of IVL to inform clinical practice guidelines and support decision making on optimal treatments and thereby improve outcomes for CLTI patients.
